# Fluvastatin promotes chondrogenic differentiation of adipose-derived mesenchymal stem cells by inducing bone morphogenetic protein 2

**DOI:** 10.1186/s40360-022-00600-7

**Published:** 2022-08-09

**Authors:** Masanari Kuwahara, Yukio Akasaki, Norio Goto, Ichiro Kurakazu, Takuya Sueishi, Masakazu Toya, Taisuke Uchida, Tomoaki Tsutsui, Ryota Hirose, Hidetoshi Tsushima, Yasuharu Nakashima

**Affiliations:** Department of Orthopaedic Surgery, Graduate School of Medical Sciences, Kyushu University, 3-1-1 Maidashi, Higashi-ku, Fukuoka city, Fukuoka, 812-8582 Japan

**Keywords:** Fluvastatin, ADMSCs, Chondrogenic differentiation, BMP2, Mevalonate pathway, GGPP, RhoA–ROCK signaling

## Abstract

**Background:**

Adipose-derived mesenchymal stem cells (ADMSCs) are a promising source of material source for medical regeneration of cartilage. Growth factors, including transforming growth factor-β (TGFβ) subfamily members and bone morphogenetic proteins (BMPs), play important roles in inducing and promoting chondrogenic differentiation of MSCs. However, these exogenous growth factors have some drawbacks related to their cost, biological half-life, and safety for clinical application. Several studies have reported that statins, the competitive inhibitors of 3-hydroxy-2-methylglutaryl coenzyme A (HMG-CoA) reductase, induce the expression of BMP2 in multiple cell types as the pleotropic effects. The objective of this study was to investigate the effects of fluvastatin during chondrogenic differentiation of human ADMSCs (hADMSCs).

**Methods:**

The effects of fluvastatin were analyzed during chondrogenic differentiation of hADMSCs in the pellet culture without exogenous growth factors by qRT-PCR and histology. For functional studies, Noggin, an antagonist of BMPs, mevalonic acid (MVA) and geranylgeranyl pyrophosphate (GGPP), metabolites of the mevalonate pathway, ROCK inhibitor (Y27632), or RAC1 inhibitor (NSC23766) were applied to cells during chondrogenic differentiation. Furthermore, RhoA activity was measured by RhoA pulldown assay during chondrogenic differentiation with or without fluvastatin. Statistically significant differences between groups were determined by Student’s t-test or the Tukey–Kramer test.

**Results:**

Fluvastatin-treated cells expressed higher levels of *BMP2*, *SOX9*, *ACAN,* and *COL2A1* than control cells, and accumulated higher levels of glycosaminoglycans (GAGs). Noggin significantly inhibited the fluvastatin-mediated upregulation of *ACAN* and *COL2A1*. Both MVA and GGPP suppressed the effects of fluvastatin on the expressions of *BMP2*, *SOX9*, *ACAN,* and *COL2A1*. Furthermore, fluvastatin suppressed the RhoA activity, and inhibition of RhoA–ROCK signaling by Y27632 increased the expressions of *BMP2*, *SOX9*, *ACAN,* and *COL2A1,* as well as fluvastatin.

**Conclusions:**

Our results suggest that fluvastatin promotes chondrogenic differentiation of hADMSCs by inducing endogenous BMP2, and that one of the mechanisms underlying the effects is inhibition of RhoA–ROCK signaling via suppression of GGPP. Fluvastatin is a safe and low-cost compound that holds promise for use in transplantation of hADMSCs for cartilage regeneration.

**Supplementary Information:**

The online version contains supplementary material available at 10.1186/s40360-022-00600-7.

## Background

Due to their high self-renewal capacity and chondrogenic differentiation potential, mesenchymal stem cells (MSCs) are a promising source of material source for medical regeneration of cartilage [1, 2]. MSCs can be isolated from various tissues such as bone marrow, adipose tissue, and synovium. Adipose-derived mesenchymal stem cells (ADMSCs) have advantages over other cells in terms of harvest volume and the safety of the procedure [3, 4]. Johnstone et al. first reported in vitro chondrogenic differentiation of MSCs in pellet culture [5], which requires growth factors and other compounds [6]. In particular, growth factors play important roles in inducing and promoting chondrogenic differentiation of MSCs, including transforming growth factor-β (TGFβ) subfamily members and bone morphogenetic proteins (BMPs) [7]. However, these exogenous growth factors have some drawbacks related to their cost, biological half-life, and safety for clinical application [8–11].

Statins are competitive inhibitors of 3-hydroxy-2-methylglutaryl coenzyme A (HMG-CoA) reductase and are used clinically throughout the world to decrease serum cholesterol levels by inhibiting the mevalonate pathway [12]. Importantly, several studies have suggested that statins have pleotropic effects, including alterations of cell differentiation, by inhibiting synthesis of isoprenoid, which is required for post-translational modification of small GTP-binding proteins such as the members of the Rho family [13].

Mundy et al. first reported that statins induced the expression of *BMP2* in osteoblasts and stimulated new bone formation in vitro and in vivo [14]. Subsequently, statins were reported to induce the expression of *BMP2* in multiple cell types, including MSCs during osteogenic differentiation [15], chondrocytes [16, 17], intervertebral disc cells [18], vascular smooth muscle cells [19], and colorectal cancer cell lines [20]. In mesenchymal cells to the chondrogenic lineage and in growth plate chondrocytes, BMP signaling stimulates chondrogenic differentiation and maturation. Among the members of this family, BMP2 plays a crucial role in the condensation of MSCs and promotes the synthesis of extracellular matrix [7, 21, 22]. Furthermore, exogenous BMP2, alone or combined with other growth factors, has been used to differentiate MSCs into the chondrogenic lineage [23–27]. Therefore, we hypothesized that statins would promote chondrogenic differentiation of MSCs by inducing endogenous BMP2.

In this study, we showed that fluvastatin could promote chondrogenic differentiation of human ADMSCs (hADMSCs) in pellet culture without exogenous growth factors, and investigated the underlying mechanism.

## Materials and methods

### Cell culture

hADMSCs were purchased from Promo Cell (Heidelberg, Germany). The cells were cultured in MSC Growth Medium 2 (Promo Cell) at 37 °C in an atmosphere containing 5% CO_2_ and maintained at sub-confluence to prevent spontaneous differentiation, according to instructions recommended by the manufacturer. Cells from passages 3–6 were used in this study.

### Cell viability assay

hADMSCs were seeded in 96-well plates at a density of 5 × 10^3^ cells/well, incubated in high-glucose GlutaMAX DMEM medium (Gibco, Langley, OK, USA) with 1% FBS supplemented with fluvastatin (Toronto Research Chemicals, North York, Canada) at 0, 0.01, 0.1 or 1 μM for 2 weeks. Culture media were replaced every 3–4 days. The cell viability assay was performed using a CellTiter-Glo Luminescent Cell Viability Assay kit (Promega, Fitchburg, WI) in accordance with the manufacturer’s protocol. Luminescence was measured using a MicroLumatPlus LB96V Microplate Luminometer (EG&G Berthold, Bad Wildbad, Germany).

### In vitro chondrogenic differentiation

hADMSCs were seeded in 96-well U-bottom plates (Sumitomo Bakelite, Tokyo, Japan) at a density of 2 × 10^5^ cells/well in high-glucose GlutaMAX DMEM medium and incubated for 48 h. Under these conditions, the cells spontaneously formed 3D pellets. Then, to induce chondrogenic differentiation, the pellets were incubated in chondrogenic medium for 2 weeks at 37 °C in an atmosphere containing 5% CO_2_. High-glucose GlutaMAX DMEM medium supplemented with L-proline (40 μg/ml) (Sigma-Aldrich, St. Louis, MO, USA), dexamethasone (0.1 μM) (Sigma-Aldrich), 1% ITS (Sigma-Aldrich), L-ascorbic acid (50 μg/ml) (Sigma-Aldrich), and sodium pyruvate (110 μg/ml) (Gibco) was used as control chondrogenic medium. Chondrogenic differentiation of hADMSCs was performed under the following three conditions: control medium, control medium with human TGFβ1 (10 ng/ml) (R&D Systems, Minneapolis, MN, USA) or fluvastatin (0.1 μM). Chondrogenic media were replaced every 3–4 days.

### Total RNA extraction and quantitative real-time polymerase chain reaction (qRT-PCR)

Total RNA was extracted from three hADMSC pellets for each condition. Briefly, pellets were crushed in a mini homogenizer and lysed in TRIzol reagent (Invitrogen, Carlsbad, CA, USA). Complementary DNA was synthesized using the PrimeScript RT Reagent (Takara Bio, Kusatsu, Japan). qRT-PCR was performed on a LightCycler 2.0 Instrument (F. Hoffmann–La Roche AG, Basel, Switzerland) using TB Green Premix EX TaqII (Takara Bio). *GAPDH* was used as an internal control and used to normalize the levels of other target genes across samples. The sequences of the forward and reverse primers were as Table 1.

**Table 1:** Primer sequences used for qRT-PCR

Genes	Forward (5’-3’)	Reverse (5’-3’)
*Human SOX9*	AGACCTTTGGGCTGCCTTAT	TAGCCTCCCTCACTCCAAGA
*Human ACAN*	AGGCAGCGTGATCCTTACC	GGCCTCTCCAGTCTCATTCTC
*Human COL2A1*	CAACACTGCCAACGTCCAGAT	CTGCTTCGTCCAGATAGGCAAT
*Human COL10A1*	ACGCTGAACGATACCAAATG	TGCTATACCTTTACTCTTTATGGTGTA
*Human BMP2*	CGAAACACAAACAGCGGAAAC	GCCACAATCCAGTCGTTCCA
*Human GAPDH*	GGTGAAGGTCGGAGTCAACGGA	GAGGGATCTCGCTCCTGGAAGA

### Alcian blue staining

Pellets of hADMSCs were differentiated under each condition for 2 weeks, fixed with 4% paraformaldehyde for 24 h, and embedded in paraffin. Sections were deparaffinized, rehydrated, and stained with 0.3% Alcian blue 8GX for 10 min. The percentages of positively stained areas were measured using the BZ-II Analyzer software (Keyence, Osaka, Japan).

### BMP inhibition experiment

Recombinant human Noggin 500 ng/mL (R&D Systems) was applied to cells in control chondrogenic medium with or without 0.1 μM fluvastatin, as mentioned above. On days 14, total RNA extraction and qRT-PCR were performed, and the expressions of *ACAN* and *COL2A1* was confirmed.

### Mevalonate pathway experiment

Mevalonic acid (100 μM) (MVA; Sigma-Aldrich) or geranylgeranyl pyrophosphate (20 μM) (GGPP; Sigma-Aldrich) was applied to cells in chondrogenic medium with 0.1 μM fluvastatin, as described above. On days 4 and 7, total RNA extraction and qRT-PCR were performed, and the expressions of *BMP2, SOX9, ACAN,* and *COL2A1* was confirmed.

### RhoA–ROCK or RAC1 signaling inhibition

Y27632 (20 μM) (R&D Systems), a ROCK inhibitor, or NSC23766 (50 μM) (Sigma-Aldrich), a RAC1 inhibitor, was applied to cells in control chondrogenic medium, as described above. On days 4 and 7, total RNA extraction and qRT-PCR were performed, and the expressions of *BMP2, SOX9, ACAN,* and *COL2A1* was confirmed.

### RhoA pulldown assay and western blotting

RhoA activity was analyzed using the Rho Activation Assay Kit (Cytoskelton, Denver, CO, USA) according to the instructions of the manufacturer. hADMSCs were seeded in 96-well U-bottom plates at a density of 2 × 10^5^ cells/well. After the cells spontaneously formed 3D pellets, the cells were induced chondrogenic differentiation with or without 0.1 μM fluvastatin. On day 1, the culture medium was replaced with fresh medium of each condition, and after 1 h, whole-cell lysates were extracted from five pellets. Briefly, pellets were crushed in a mini homogenizer and lysed using lysis buffer. Then, the cell lysates were clarified by centrifugation and incubated with rhotekin-Rho binding domain (RBD) beads at 4 °C for 1 h to selectively precipitate GTP-RhoA, the active form RhoA. Whole-cell lysates and the precipitates were determined the amount of RhoA by western blotting. The cell lysates were electrophoresed in 4–12% gradient polyacrylamide gels (Invitrogen), and the resolved proteins were transferred to nitrocellulose membranes (Amersham Biosciences, Arlington Heights, IL, USA). Membranes were blocked with blocking buffer (Takara Bio), washed in TBS with Tween (TBST), and incubated with primary antibodies against RhoA (number ARH05; Cytoskelton) and GAPDH (number 5174; Cell Signaling Technology, Danvers, MA, USA) diluted in Can Get Signal Immunoreaction Enhancer Solution 1 (TOYOBO, Osaka, Japan) (RhoA, 1:500; GAPDH, 1:1000) at 4 °C overnight. After the membranes were washed in TBST, secondary anti-mouse IgG antibodies (sc-516102; Santa Cruz Biotechnology, Dallas, TX, USA) or anti-rabbit IgG antibodies (number 7074; Cell Signaling Technology) were added. Immunoreactivity was detected with ECL Prime (Amersham Biosciences) and imaged on an Ez Capture MG (ATTO, Tokyo, Japan). Band densities were calculated using CS Analyzer 3.0 (ATTO).

### Statistical analysis

All experiments were repeated at least three times. Data are presented as means ± standard deviation. Statistically significant differences between groups were determined by Student’s t-test or the Tukey–Kramer test. All data analyses were performed using the JMP 15 statistical software (SAS Institute, Cary, NC, USA). *p* < 0.05 was considered statistically significant.

## Results

### Fluvastatin promotes *SOX9*, *ACAN* and *COL2A1* expressions during chondrogenic differentiation of hADMSCs (Fig. 1)

**Fig. 1:**
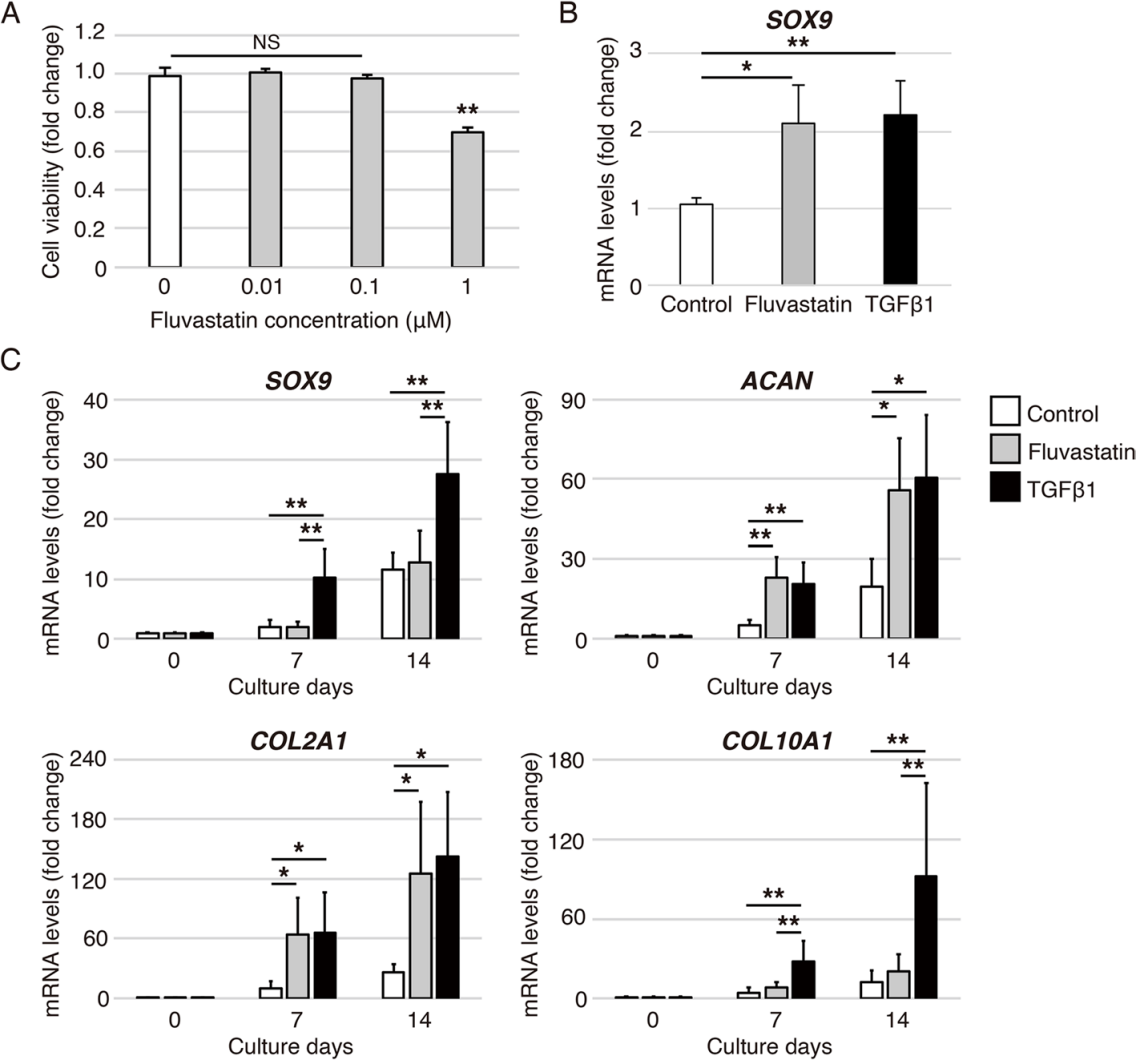
Effects of fluvastatin during chondrogenic differentiation of hADMSCs. **A** Effects of fluvastatin on cell viability of hADMSCs. hADMSCs were treated with fluvastatin at 0, 0.01, 0.1 or 1 μM for 2 weeks. Cell viability is shown relative to the level on treatment with 0 μM fluvastatin. Error bars denote the means ± standard deviation (*n* = 5). ^**^p < 0.01, NS: not significant by Tukey–Kramer test. **B** Relative *SOX9* mRNA levels on day 4 during chondrogenic differentiation of hADMSCs assessed by qRT-PCR; control, fluvastatin, and TGFβ1 groups are shown. Gene expression is given relative to the level in the control group. Error bars denote means ± standard deviation (*n* = 4). ^*^p < 0.05, ^**^p < 0.01 by Tukey–Kramer test. **C** Effects of fluvastatin on gene expression during chondrogenic differentiation of hADMSCs, as determined by qRT-PCR. Relative mRNA levels of *SOX9*, *ACAN*, *COL2A1*, and *COL10A1* in chondrogenic differentiation of hADMSCs on days 0, 7, and 14 among control, fluvastatin, and TGFβ1 groups. Gene expression at each stage is shown relative to the level on day 0. Error bars denote the means ± standard deviation (*n* = 5). ^*^p < 0.05, ^**^p < 0.01 by Tukey–Kramer test

First, we investigated the effect of fluvastatin on cell viability of hADMSCs. Incubation with fluvastatin at concentrations below 0.1 μM for 2 weeks did not affect cell viability of hADMSCs (Fig. 1A).

Next, to examine whether fluvastatin promotes chondrogenic differentiation of hADMSCs, the following three conditions were prepared: the control, fluvastatin, and TGFβ1. TGFβ1, a major inducer of chondrogenic differentiation, was used as a positive control. The expressions of *SOX9, ACAN,* and *COL2A1* as chondrogenic differentiation markers, and *COL10A1* as a hypertrophic differentiation marker, were measured. The expression of *SOX9* was significantly higher in the TGFβ1 and fluvastatin groups than in the control group on day 4 (Fig. 1B). *SOX9* expression in the TGFβ1 group on days 7 and 14 was significantly higher than in the other groups (Fig. 1C). The expressions of *ACAN* and *COL2A1* were significantly higher in the TGFβ1 and fluvastatin groups than in the control group on days 7 and 14. There were no significant differences in *ACAN* and *COL2A1* expression between the TGFβ1 and fluvastatin groups (Fig. 1C). The expression of *COL10A1* was significantly higher in the TGFβ1 group than in the other groups on days 7 and 14 (Fig. 1C).

### Fluvastatin increases staining of glycosaminoglycans in pellets of hADMSCs

**Fig. 2:**
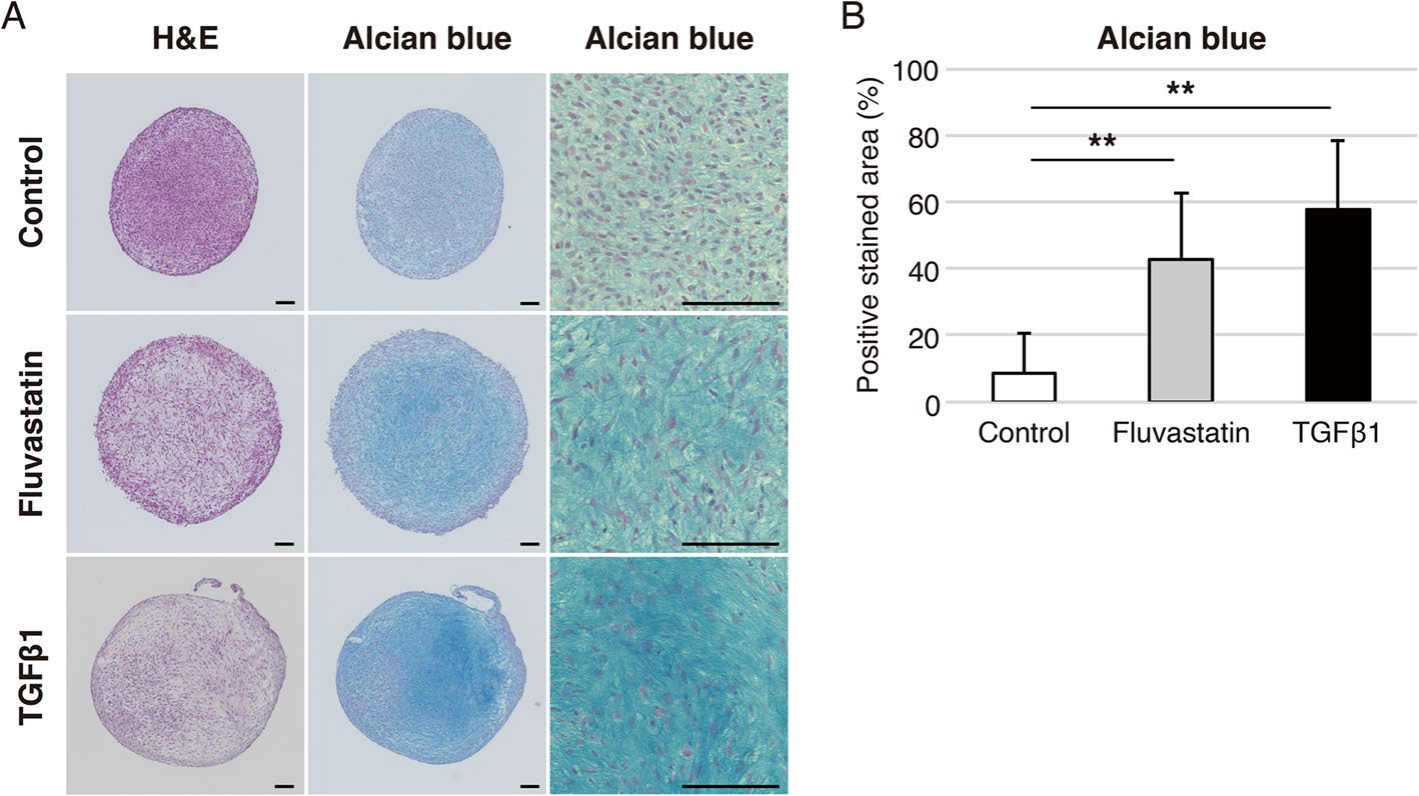
Histological appearance of pellets of hADMSCs cultured in chondrogenic medium for 14 days. **A** Histological appearance of pellets of hADMSCs stained with hematoxylin and eosin (H&E) and Alcian blue; control, fluvastatin, and TGFβ1 groups are shown. Bars represent 100 μm. **B** Percentages of Alcian blue–positive areas in the control, fluvastatin, and TGFβ1 groups. Error bars denote means ± standard deviation (*n* = 10). ^**^p < 0.01 by Tukey–Kramer test

After 14 days of chondrogenic differentiation under the three conditions, we evaluated accumulation of glycosaminoglycans (GAGs) in pellets stained with Alcian blue. Levels of GAGs were significantly higher in the TGFβ1 and fluvastatin groups, but did not differ significantly between the TGFβ1 and fluvastatin groups (Fig. 2A and B).

### Fluvastatin induces *BMP2* expression in chondrogenic differentiation of hADMSCs

**Fig. 3:**
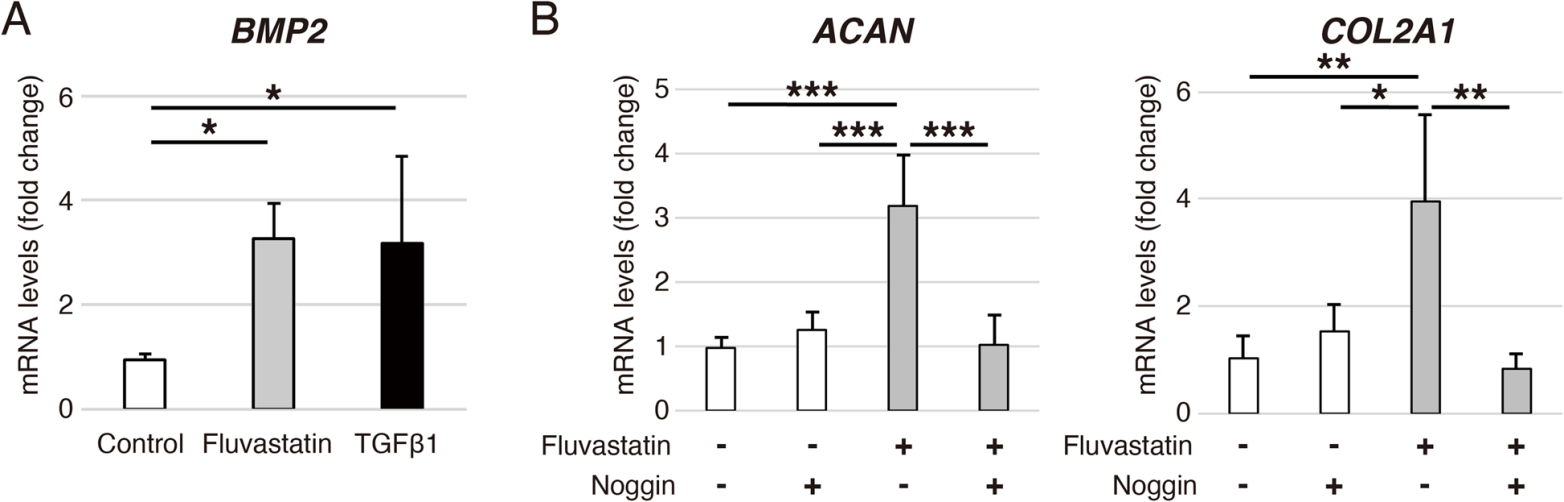
Fluvastatin-induced *BMP2* expression and its effect on chondrogenic differentiation of hADMSCs. **A** Relative *BMP2* mRNA levels on day 4 during chondrogenic differentiation of hADMSCs assessed by qRT-PCR; control, fluvastatin, and TGFβ1 groups are shown. Gene expression is given relative to the level in the control group. Error bars denote means ± standard deviation (*n* = 4). ^*^p < 0.05 by Tukey–Kramer test. **B** Effects of Noggin on gene expression promoted by fluvastatin in chondrogenic differentiation of hADMSCs assessed by qRT-PCR. Relative *ACAN* and *COL2A1* mRNA levels on day 14 in chondrogenic differentiation of hADMSCs cultured with or without fluvastatin or Noggin. Gene expression is expressed relative to the level in cells cultured without fluvastatin or Noggin. Error bars denote means ± standard deviation (*n* = 4). ^*^p < 0.05, ^**^p < 0.01, ^***^p < 0.001 by Tukey–Kramer test

To elucidate the molecular mechanism of fluvastatin-promoted chondrogenic differentiation of hADMSCs, we confirmed the expression of *BMP2*. The results revealed that expression of *BMP2* on day 4 during chondrogenic differentiation of hADMSCs was significantly higher in the TGFβ1 and fluvastatin groups than in the control group. Again, there was no significant difference between the TGFβ1 and fluvastatin groups (Fig. 3A).

### Noggin blocks the expressions of *ACAN* and *COL2A1* promoted by fluvastatin in chondrogenic differentiation of hADMSCs (Fig. 3)

To determine whether BMP2 induced by fluvastatin promotes the expressions of *ACAN* and *COL2A1* during chondrogenic differentiation of hADMSCs, we administered 500 ng/mL Noggin to cells. In chondrogenic medium containing fluvastatin, Noggin significantly blocked the elevation of *ACAN* and *COL2A1* expression on day 14 (Fig. 3B). On the other hand, Noggin did not suppress the baseline chondrogenic differentiation in control medium without fluvastatin (Fig. 3B).

### Mevalonic acid and geranylgeranyl pyrophosphate suppress the effects of fluvastatin in chondrogenic differentiation of hADMSCs

**Fig. 4:**
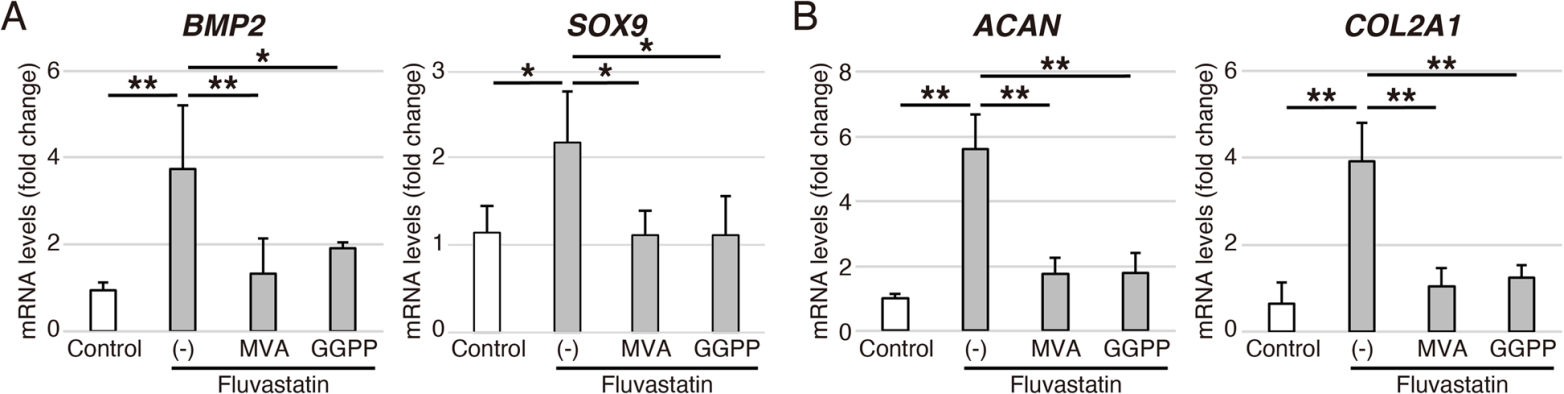
Effects of MVA or GGPP on fluvastatin-induced gene expression by during chondrogenic differentiation of hADMSCs. **A** Relative *BMP2* and *SOX9* mRNA levels on day 4 and **B** relative *ACAN* and *COL2A1* mRNA levels on day 7 in chondrogenic differentiation of hADMSCs cultured with or without fluvastatin, MVA, or GGPP, as determined by qRT-PCR. Gene expression is expressed relative to the level in cells cultured without fluvastatin, MVA, or GGPP. Error bars denote means ± standard deviation (*n* = 4). ^*^p < 0.05, ^**^p < 0.01 by Tukey–Kramer test

In the mevalonate pathway, MVA is produced from HMG-CoA by HMG-CoA reductase, and subsequently metabolized to GGPP [28]. Hence, for the further mechanistic analyses, we administered 100 μM MVA or 20 μM GGPP to cells in chondrogenic medium with fluvastatin. Both MVA and GGPP significantly suppressed the expressions of *BMP2* and *SOX9* on day 4 (Fig. 4A), and the expressions of *ACAN* and *COL2A1* on day 7 (Fig. 4B).

### Inhibition of RhoA–ROCK signaling increases *BMP2* and *SOX9* expressions followed by *ACAN* and *COL2A1* during chondrogenic differentiation of hADMSCs

**Fig. 5:**
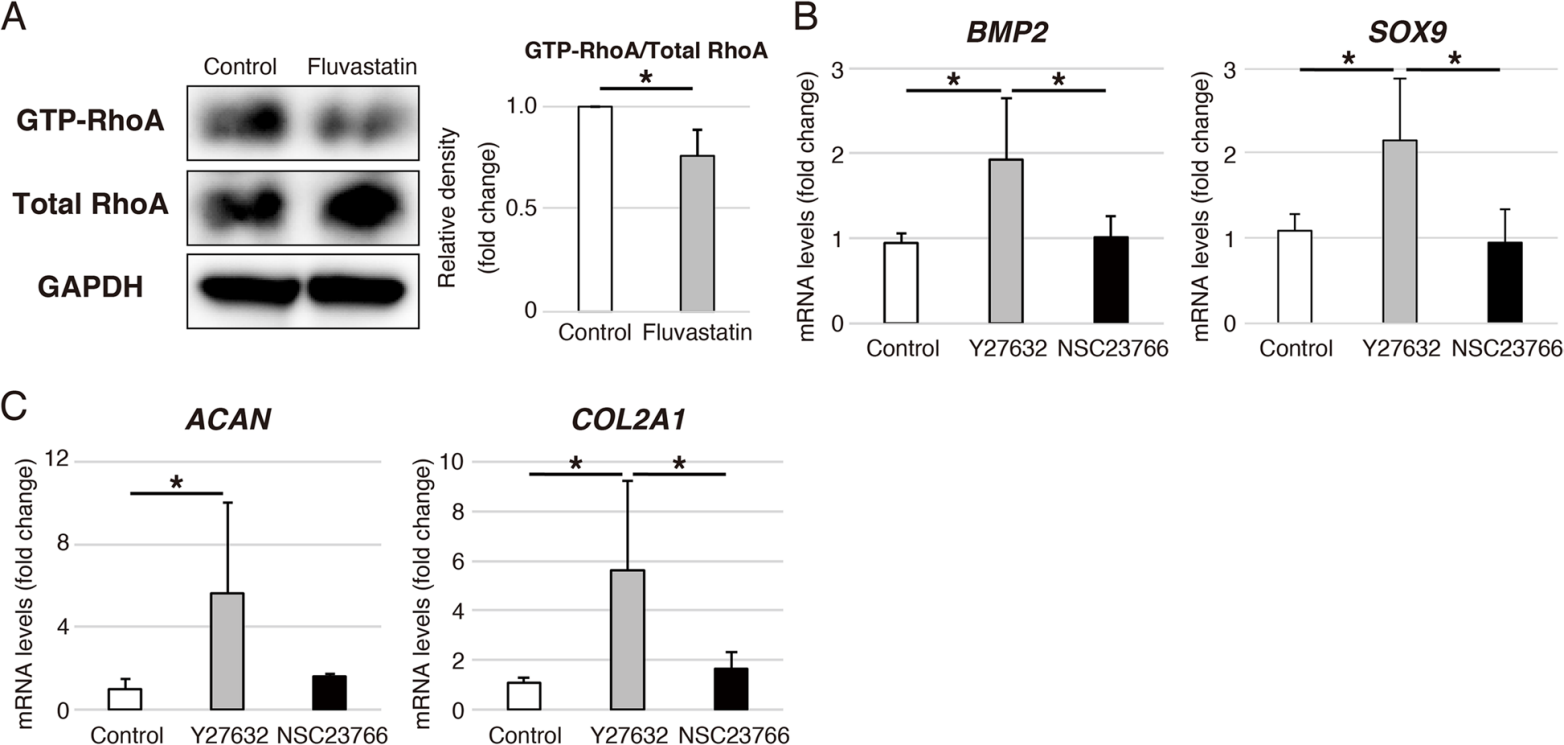
Effects of fluvastatin on the RhoA activity in chondrogenic differentiation of hADMSCs. **A** Levels of GTP-RhoA, total RhoA, and GAPDH on day 1 in chondrogenic differentiation of hADMSCs cultured with or without fluvastatin, as determined by western blotting. GAPDH was used as a loading control. These blot images were cropped. Error bars denote means ± standard deviation (*n* = 3). ^*^p < 0.05 by Student’s t-test. **B** Relative *BMP2* and *SOX9* mRNA levels on day 4 and (**C**) relative *ACAN* and *COL2A1* mRNA levels on day 7 in chondrogenic differentiation of hADMSCs cultured with Y27632 or NSC23766, as determined by qRT-PCR. Gene expression is expressed relative to the level in cells cultured without Y27632 or NSC23766. Error bars denote means ± standard deviation (*BMP2* and *SOX9*, *n* = 4; *ACAN* and *COL2A1*, *n* = 5). ^*^p < 0.05 by Tukey–Kramer test

The GTP-RhoA, the active form RhoA, in cells treated with fluvastatin was significantly decreased compared to control on day 1 during chondrogenic differentiation of hADMSCs (Fig. 5A). Next, we applied 20 μM ROCK inhibitor (Y27632) and 50 μM RAC1 inhibitor (NSC23766) to cells in control chondrogenic medium in order to investigate the inhibitory effect of RhoA–ROCK or RAC1 signaling. ROCK inhibitor, but not RAC1 inhibitor, increased the expressions of *BMP2* and *SOX9* on day 4 (Fig. 5B), and the expression of *ACAN* and *COL2A1* on day 7 (Fig. 5C).

## Discussion

In the present study, we revealed the positive effects of fluvastatin on chondrogenic differentiation of hADMSCs in the absence of exogenous growth factors. Fluvastatin significantly induced *BMP2*, *SOX9*, *ACAN* and *COL2A1* expressions and accumulated GAGs in the pellet of hADMSCs. In the functional analyses, BMP inhibition by Noggin significantly inhibited the fluvastatin-mediated upregulation of *ACAN* and *COL2A1*. Exogenously added MVA and GGPP, HMG-CoA products, reversed the effects of fluvastatin on the gene expressions. Fluvastatin suppressed the RhoA activity, and inhibition of RhoA–ROCK signaling by Y27632 increased *BMP2*, *SOX9*, *ACAN,* and *COL2A1* expressions. Taken together, our findings suggest that fluvastatin promotes chondrogenic differentiation of hADMSCs by inducing endogenous BMP2.

Statins were previously reported to promote chondrogenic differentiation of ATDC5 cells, an in vitro model of chondrogenic differentiation, and to rescue the chondrogenic differentiation ability of induced pluripotent stem (iPS) cells from patients with achondroplasia or thanatophoric dysplasia [29, 30]. These studies proposed that the mechanism by which statins influence chondrogenic differentiation is related to the Indian Hedgehog pathway or fibroblast growth factor receptor 3 (FGFR3); however, it is not fully understood. In this study, we focused on BMP2 as a statin-induced growth factor [15–20]. Exogenous BMP2 promotes chondrogenic differentiate of MSCs [23–27], and endogenous BMP2 expression is elevated during the early stage of TGFβ1-induced chondrogenic differentiation of MSCs [26, 31]. As we hypothesized, fluvastatin promoted the chondrogenic differentiation in hADMSCs accompanied by elevated *BMP2* expression. This is the first to elucidate the potential of fluvastatin on chondrogenic induction of MSCs as alternative to exogenous growth factors for clinical application.

Statins exert the pleotropic effects by inhibiting isoprenoid synthesis, which is required for post-translational modification of small GTP-binding proteins such as the members of the Rho family [13]. In fact, fluvastatin decreased the RhoA activity, a downstream effector of GGPP, one of the isoprenoids, and the effects of fluvastatin were abolished by exogenous GGPP addition. Furthermore, the chemical inhibition of RhoA–ROCK signaling mimicked the anabolic effects of fluvastatin on chondrogenic differentiation of hADMSC. Several previous studies reported that the inhibition of RhoA–ROCK signaling enhanced chondrogenic gene expressions and cartilaginous extracellular matrix accumulation by reducing cytoskeletal tension in MSCs pellet culture [32–34]. Therefore, the underlying mechanism of the effects of fluvastatin might be involved in the inhibition of RhoA–ROCK signaling and the subsequent cytoskeletal reorganization.

In this study, the expression of *SOX9*, a master regulator of chondrogenesis, was higher on day 4 in the fluvastatin group than in the control group, after that, there was no significant difference between the two groups. In general, SOX9 is a transcription factor that acts during the early phase of chondrogenic differentiation of MSCs [35, 36]. BMP2 signaling and cytoskeletal reorganization, which we propose to be the mechanism by which fluvastatin promotes chondrogenesis, have been reported to be closely involved in SOX9 expression. BMP2 signaling increases the level of SOX9 expression by the p38 MAPK pathway within half a day after BMP2 administration to mouse embryonic fibroblasts [37]. The cytoskeletal reorganization of MSCs by the continuous low-intensity ultrasound stimulation also induces SOX9 upregulation immediately via the phosphorylation of ERK1/2 [38]. Based on these findings, the impact of fluvastatin on SOX9 expression should be predominant in the early stage of chondrogenic differentiation of hADMSCs.

This study has several limitations. First, we used primary hADMSCs, which may differ by donor. Second, we conducted only in vitro studies of chondrogenic differentiation, therefore we need to confirm the effects of fluvastatin in vivo. Third, this study failed to show that fluvastatin directly inhibits the intracellular synthesis of MVA and GGPP during chondrogenic differentiation of hADMSCs, because reliable microanalysis could not be performed under pellet culture. However, a number of reports used exogenous MVA and GGPP in the rescue experiment to abolish the effect of statin [39–41]. In addition, fluvastatin is a lipophilic statin with capable of transferring into a cell. Hence, fluvastatin exogenously administered could surely function in cytoplasm to inhibit the intracellular synthesis of MVA and GGPP.

## Conclusions

In summary, we demonstrated that fluvastatin promotes chondrogenic differentiation of hADMSCs by inducing endogenous BMP2. One of the mechanisms underlying the effects of fluvastatin administration effects was inhibition of RhoA–ROCK signaling via suppression of GGPP. Although more work is needed to elucidate its molecular mechanism, fluvastatin is a safe and low-cost compound that hold promise for use in transplantation of hADMSCs for cartilage regeneration.

## Supplementary Information

**Additional file 1. Fig. S1 **The original uncropped blot in Figure 5A. Black-boxed areas in the original blot were included in the main paper.

## Data Availability

The datasets used and/or analysed during the current study available from the corresponding author on reasonable request.

## Abbreviations

MSCs: Mesenchymal stem cells
hADMSCs: human adipose-derived mesenchymal stem cells
TGFβ: transforming growth factor β
BMPs: bone morphogenetic proteins
HMG-CoA: 3-hydroxy-2-methylglutaryl coenzyme A
DMEM: Dulbecco’s modified Eagle’s medium
FBS: fetal bovine serum
3D: three-dimensional
ITS: insulin–transferrin–selenium
RT: reverse transcription
qRT-PCR: quantitative real-time polymerase chain reaction
GAPDH: glyceraldehyde-3-phosphate dehydrogenase
SOX9: SRY-box transcription factor 9
ACAN: aggrecan
COL2A1: collagen type II alpha 1 chain
COL10A1: collagen type X alpha 1 chain
GAGs: glycosaminoglycans
MVA: mevalonic acid
GGPP: geranylgeranyl pyrophosphate
RhoA: ras homolog family member A
ROCK: Rho associated coiled-coil containing protein kinase
RAC1: Rac family small GTPase 1
iPS cells: induced pluripotent stem cells
FGFR3: fibroblast growth factor receptor 3
MAPK: mitogen-activated protein kinase
