# Supplementary material for: Fluvastatin promotes chondrogenic differentiation of adipose-derived mesenchymal stem cells by inducing bone morphogenetic protein 2
Source: BMC Pharmacol Toxicol. 2022 Aug 9;23:61. doi: 10.1186/s40360-022-00600-7 (PMC9361648; doi:10.1186/s40360-022-00600-7)

**Fig. S1** The original uncropped blot in Figure 5A. Black-boxed areas in the original blot were included in the main paper.


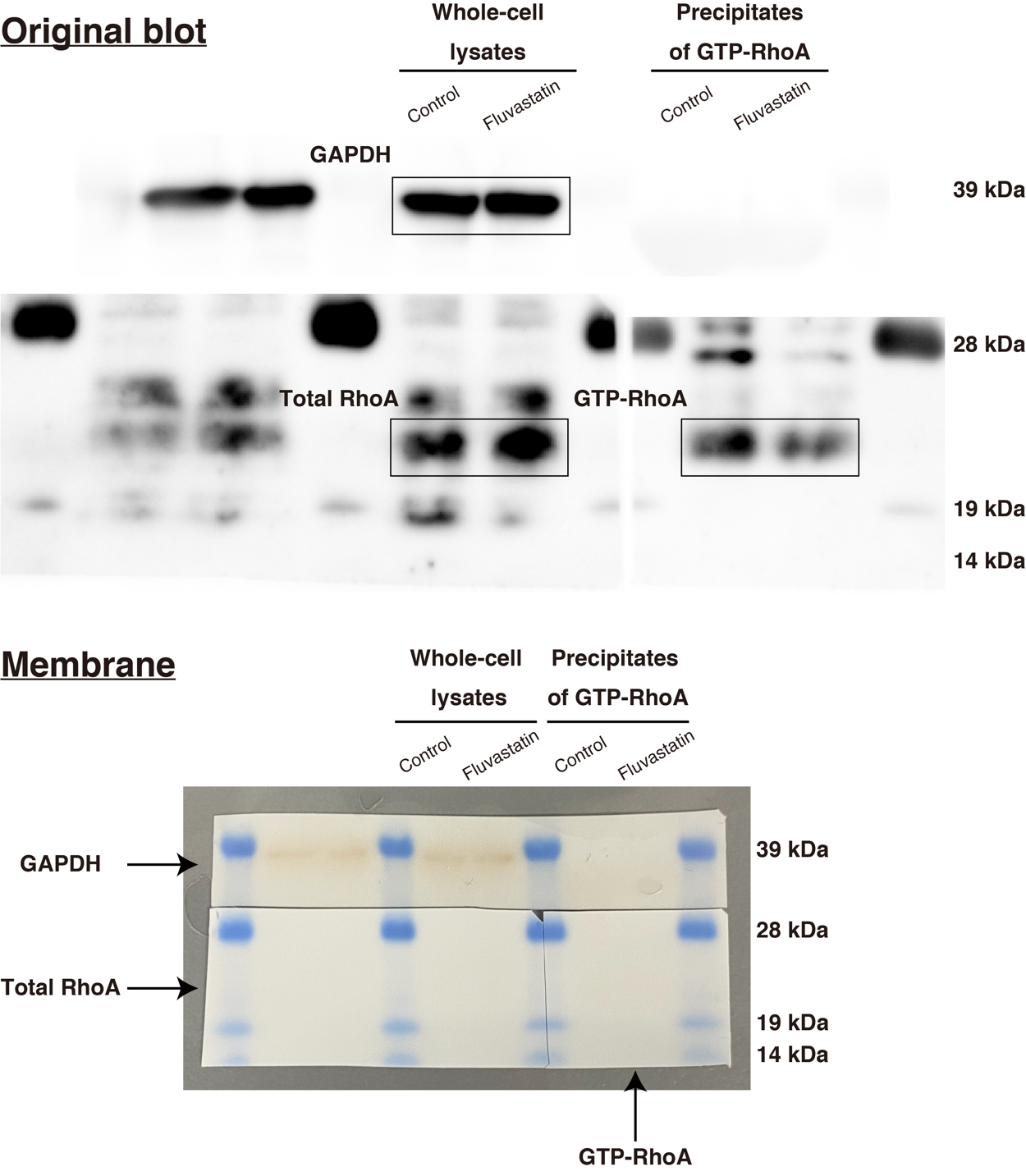

Supplement: Supplementary file 1 — Additional file 1. Fig. S1 The original uncropped blot in Figure 5A. Black-boxed areas in the original blot were included in the main paper. [file 40360_2022_600_MOESM1_ESM.docx]
